# Early-Life Exposure to Perfluoroalkyl Substances and Childhood Metabolic Function

**DOI:** 10.1289/EHP303

**Published:** 2016-09-02

**Authors:** Abby F. Fleisch, Sheryl L. Rifas-Shiman, Ana M. Mora, Antonia M. Calafat, Xiaoyun Ye, Heike Luttmann-Gibson, Matthew W. Gillman, Emily Oken, Sharon K. Sagiv

**Affiliations:** 1 Division of Endocrinology, Boston Children’s Hospital, Boston, Massachusetts, USA; 2 Obesity Prevention Program, Department of Population Medicine, Harvard Medical School and Harvard Pilgrim Health Care Institute, Boston, Massachusetts, USA; 3 Department of Environmental Health, Boston University School of Public Health, Boston, Massachusetts, USA; 4 Central American Institute for Studies on Toxic Substances (IRET), Universidad Nacional, Heredia, Costa Rica; 5 Division of Laboratory Sciences, National Center for Environmental Health, Centers for Disease Control and Prevention, Atlanta, Georgia, USA; 6 Department of Environmental Health, and; 7 Department of Nutrition, Harvard School of Public Health, Boston, Massachusetts, USA; 8 Center for Environmental Research and Children’s Health (CERCH), School of Public Health, University of California, Berkeley, Berkeley, California, USA

## Abstract

**Background:**

Perfluoroalkyl substances (PFASs) are synthetic chemicals that may persist in the environment and in humans. There is a possible association between early-life PFAS exposure and metabolic dysfunction in later life, but data are limited.

**Methods:**

We studied 665 mother–child pairs in Project Viva, a Boston, Massachusetts-area cohort recruited 1999–2002. We quantified concentrations of PFASs [perfluorooctanoate (PFOA), perfluorooctane sulfonate (PFOS), perfluorononanoate (PFNA), perfluorohexane sulfonate (PFHxS), and perfluorodecanoate (PFDeA)] in maternal plasma collected at the first prenatal visit (median, 9.6 weeks gestation) and in child plasma from the mid-childhood research visit (median, 7.7 years). We assessed leptin, adiponectin, and homeostatic model assessment of insulin resistance (HOMA-IR) in mid-childhood. We fit covariate-adjusted linear regression models and conducted stratified analyses by child sex.

**Results:**

Children with higher PFAS concentrations had lower HOMA-IR [e.g., –10.1% (95% CI: –17.3, –2.3) per interquartile range increment in PFOA]. This inverse association between child PFAS and HOMA-IR was more pronounced in females [e.g., PFOA: –15.6% (95% CI: –25.4, –4.6) vs. –6.1% (95% CI: –16.2, 5.2) for males]. Child PFAS plasma concentrations were not associated with leptin or adiponectin. Prenatal PFAS plasma concentrations were not associated with leptin, adiponectin, or HOMA-IR in offspring.

**Conclusions:**

We found no evidence for an adverse effect of early-life PFAS exposure on metabolic function in mid-childhood. In fact, children with higher PFAS concentrations had lower insulin resistance.

**Citation:**

Fleisch AF, Rifas-Shiman SL, Mora AM, Calafat AM, Ye X, Luttmann-Gibson H, Gillman MW, Oken E, Sagiv SK. 2017. Early-life exposure to perfluoroalkyl substances and childhood metabolic function. Environ Health Perspect 125:481–487; http://dx.doi.org/10.1289/EHP303

## Introduction

Perfluoroalkyl substances (PFASs) are synthetically produced compounds used as additives in clothing, furniture, carpets, and cookware to make the items nonstick and stain repellant (Lindstrom et al. 2011). Long-chain PFASs persist in the environment and in humans with a half-life of 3–5 years (Olsen et al. 2007). Also, several PFASs are ubiquitous and detectable in varying concentrations in almost all U.S. children and adults (Calafat et al. 2007; CDC 2015).

PFASs have structural homology with fatty acids and may have endocrine-disrupting properties. A growing body of literature suggests that PFAS exposure may contribute to metabolic dysfunction (Audouze et al. 2013; U.S. EPA 2013) through up-regulation of fatty acid oxidation pathways (Guruge et al. 2006; Hu et al. 2005) and concomitantly increased oxidative stress (Karpe et al. 2011). However, PFASs also function as peroxisome proliferator–activated receptor (PPAR) agonists (Vanden Heuvel et al. 2006), which would be expected to improve, rather than exacerbate, insulin resistance.

The epidemiologic literature is in line with these conflicting mechanisms of action. Some (Lin et al. 2009; Lind et al. 2014; Timmermann et al. 2014) but not all (Fisher et al. 2013; MacNeil et al. 2009; Nelson et al. 2010) cross-sectional studies have linked PFAS burden with insulin resistance in adults and children. PFASs cross the placenta (Inoue et al. 2004); and in rodent models (Hines et al. 2009; Lv et al. 2013) and one prospective, population-based cohort study (Halldorsson et al. 2012), prenatally exposed offspring had greater metabolic dysfunction in adulthood. Thus, the relationship between PFASs and metabolic risk remains unclear.

In the present analysis, we evaluated the extent to which PFAS concentrations in prenatal and mid-childhood plasma were associated with biochemical markers of metabolic function in children from a Boston, Massachusetts-area birth cohort. Based on the existing literature linking prenatal PFAS exposure with adverse metabolic profiles, we hypothesized that higher prenatal and mid-childhood plasma PFAS concentrations would be associated with metabolic dysfunction, as manifest by higher leptin, lower adiponectin, and higher homeostatic model assessment of insulin resistance (HOMA-IR), in children.

## Methods

### Study Population and Design

Pregnant women were recruited to Project Viva, a prospective cohort study of prenatal exposures and offspring health, from 1999 through 2002 during their first prenatal visit (median, 10 weeks gestation) at Atrius Harvard Vanguard Medical Associates, a multi-specialty group practice in Eastern Massachusetts (Oken et al. 2015). Of 2,128 live singleton offspring, 1,116 (52.4%) children attended a mid-childhood follow-up visit (median age, 7.7 years), and 667 (31.3%) had a blood draw during that visit with measurement of at least one metabolic biomarker. Of these 667 children, 665 (99.7%) had PFAS measurements (536 with PFASs measured in 1999–2002 maternal plasma and 643 with PFASs measured in 2007–2010 child plasma) (see Figure S1). Mothers of children included in these analyses (*n* = 665) versus those excluded (*n* = 1,463) were more likely to be multiparous, have lower plasma PFAS concentrations, and were more likely to live in a census tract with lower median household income and higher percent below poverty. Their children were more likely to be black or other race/ethnicity (see Table S1).

We obtained written informed consent from mothers at each study visit and child verbal assent at the mid-childhood visit. Institutional review boards of participating institutions approved the study. The involvement of the Centers for Disease Control and Prevention (CDC) did not constitute engagement in human subject research.

### Exposure and Outcome Measurements

We measured concentrations of perfluorooctanoate (PFOA), perfluorooctane sulfonate (PFOS), perfluorononanoate (PFNA), perfluorohexane sulfonate (PFHxS), and perfluorodecanoate (PFDeA) in plasma collected from mothers in early pregnancy [median (interquartile range; IQR) 9.6 (2.1) weeks gestation] and children in mid-childhood [median (IQR), 7.7 (1.0) years of age], as previously described (Sagiv et al. 2015). Staff at the Division of Laboratory Sciences at the CDC (Atlanta, GA) quantified PFASs using on-line solid-phase extraction coupled to isotope dilution high performance liquid chromatography mass spectrometry. We measured total concentrations of each PFAS in prenatal plasma in 2013. Subsequently, studies linked specific PFAS isomers to health outcomes (Jiang et al. 2014; Yu et al. 2015). Thus, when we measured PFAS concentrations in mid-childhood plasma in 2015, we separately measured linear and branched isomers of PFOA [n-PFOA and the sum of perfluoromethylheptanoic and perfluorodimethylhexanoic acids (Sb-PFOA)] and PFOS [n-PFOS, sum of perfluoromethylheptane sulfonates (Sm-PFOS), and sum of perfluorodimethylhexane sulfonates (Sm2-PFOS)] which we summed to obtain total PFOA and PFOS concentrations. The limit of detection (LOD) was 0.1 ng/mL for all PFASs except for PFOS concentration in prenatal plasma (LOD = 0.2 ng/mL). We replaced values below the LOD with the LOD divided by the square root of 2.

We assessed metabolic function in mid-childhood through serum concentrations of leptin (marker of adiposity) and adiponectin (increases insulin sensitivity and decreases body weight) (Tilg and Moschen 2006) which we measured by radioimmunoassay (Linco Research, St. Charles, MO). We measured fasting insulin with an electrochemiluminescence immunoassay (Roche Diagnostics, Indianapolis, IN) and fasting glucose enzymatically. We estimated insulin resistance by calculating the HOMA-IR as [fasting glucose (mg/dL) × fasting insulin (mU/L))/405].

### Covariates

We collected data on maternal age, parity, smoking habits, education, partner education, household income, and marital status using questionnaires at study enrollment. We also assessed maternal glomerular filtration rate (GFR) and plasma volume expansion which increase during pregnancy and are associated with lower PFAS plasma concentrations. To calculate GFR (mL/min/1.73 m^2^), we measured creatinine in the same prenatal blood samples used for quantification of PFASs and used the Cockroft–Gault formula [GFR-CG = (140-age) × weight (kg) × 1.04/serum creatinine (μmol/L)] (Cockcroft and Gault 1976). To assess maternal plasma volume expansion, we *a*) recorded the week of gestation during which the prenatal plasma sample was obtained, and *b*) measured plasma albumin in the same prenatal blood samples used for PFAS quantification. Albumin, in addition to being an indicator for plasma volume expansion during pregnancy, is also a major PFAS binding protein (D’eon et al. 2010) and can be modeled in the same way as lipids in analyses of lipophilic compounds, such as organochlorines (Gaskins and Schisterman 2009).

We abstracted infant sex and date of delivery from medical records. We collected data on child race/ethnicity, breastfeeding duration, fast food intake, soda intake, physical activity, screen time, passive smoke exposure, and household income using questionnaires during childhood. We obtained median annual household income and percent of households below the poverty threshold for the mother’s residential census tract at the time of enrollment and in mid-childhood from 2000 U.S. Census data (U.S. Census Bureau 2000).

### Statistical Analyses

In linear regression analyses, we examined associations of prenatal and mid-childhood PFAS plasma concentrations with leptin, adiponectin, and HOMA-IR in mid-childhood. We *a priori* decided to only consider PFASs with > 65% of detectable values, which included each PFAS (PFOA, PFOS, PFNA, PFHxS, and PFDeA) at each time point (prenatal and childhood), except for prenatal plasma PFDeA (43.5% detectable values).

We ln-transformed serum concentrations of leptin, adiponectin, and HOMA-IR to meet model assumptions. For ease of interpretation, we exponentiated regression coefficients and reported results as a percent change [% change = (exp(beta) – 1) × 100].

We expressed continuous associations per IQR increment in exposure. To evaluate nonlinearity and assess outlier influence, we modeled PFAS concentrations in quartiles and fit penalized spline generalized additive models.

We accounted for covariates potentially associated with PFAS plasma concentration (Sagiv et al. 2015) and/or metabolic function (Kimbro et al. 2007; Perng et al. 2014) in all of our final models. These covariates included maternal age at enrollment (continuous), maternal education (with or without college degree), child age at mid-childhood visit (continuous), child sex (dichotomous), child race/ethnicity (white, black, Asian, Hispanic, other), census-tract median household income (continuous), and census-tract percent below poverty (continuous). In analyses of prenatal PFASs, we additionally accounted for parity (nulliparous or multiparous), maternal smoking habits (smoked during pregnancy, formerly smoked, never smoked), and week of gestation of PFAS measurement, because these covariates are potentially related to prenatal but not mid-childhood PFAS plasma concentrations. In all analyses, we substituted maternal race/ethnicity for child race/ethnicity for the 10% of participants missing data on this covariate, which resulted in > 99% of participants having race/ethnicity data.

We considered but did not include several variables in our final models that did not confound the exposure–outcome relationship (i.e., the estimate for the primary exposure changed by < 10%) or did not importantly change the results. These variables included maternal breastfeeding duration, marital status, and serum albumin and GFR (in analyses of prenatal PFASs), household income, partner education, and child fast-food intake, soda intake, physical activity, screen time, and passive smoke exposure (in analyses of child PFASs). We performed complete case analyses excluding those with missing covariates, because complete covariate information was available for 98% of participants with available exposure/outcome data.

Because prior studies have shown more pronounced associations between early-life PFAS exposure and insulin resistance in females (Halldorsson et al. 2012), we assessed for effect modification by child sex, via an interaction term and stratification.

To further investigate the inverse association between child PFAS plasma concentrations and HOMA-IR, we included all PFASs (PFOA, PFOS, PFNA, PFHxS, and PFDeA) in the same covariate-adjusted model. Because insulin resistance increases during puberty (Vryonidou et al. 2015), we restricted to prepubertal participants (70% of full cohort) which allowed us to evaluate whether a PFAS-delayed puberty association (Lopez-Espinosa et al. 2011) was driving the inverse association between child PFAS plasma concentration and HOMA-IR. We considered participants to be prepubertal if their parents reported absence of body hair growth (boys and girls), voice deepening (boys), facial hair growth (boys), and breast development (girls) [i.e., a subset of validated pubertal development scale questions (Carskadon and Acebo 1993)].

In a final sensitivity analysis, we examined the extent to which different isomers of PFAS were associated with childhood metabolic profile. We considered all isomer concentrations with > 65% detectable values (n-PFOA, n-PFOS, Sm-PFOS; see Table S2).

For penalized spline generalized additive models, we used R (version 3.0.0; R Project for Statistical Computing), and for all other analyses, we used SAS version 9.3 (SAS Institute Inc.).

## Results

### Population Characteristics

Median (IQR) maternal age at the time of prenatal enrollment was 32.5 (7.1) years; 42% of mothers were nulliparous and 64% were college graduates. Fifty-nine percent of children were white. At the mid-childhood follow-up visit [median (IQR) age, 7.7 (1.0) years], median (IQR) leptin was 3.3 (3.9) ng/mL, adiponectin 14.0 (9.7) μg/mL, and HOMA-IR 1.5 (1.3) (Table 1).

**Table 1:** Participant characteristics overall (*n *= 665 in analytic data set)*^a^* and by prenatal PFOA plasma concentration (*n *= 536 participants in analytic data set with measurement of prenatal PFOA) [median (IQR) or %].

Characteristic	Overall *n *= 665	Quartiles^*b*^ of prenatal PFOA			
		Q1 (lowest) *n *= 151	Q2 *n *= 147	Q3 *n *= 123	Q4 (highest) *n *= 115
Maternal characteristics
Age at enrollment (years)	32.5 (7.1)	33.9 (6.3)	31.9 (6.6)	32.0 (7.7)	31.2 (7.7)
Prepregnancy BMI (kg/m^2^)	23.7 (5.9)	23.6 (6.4)	23.8 (5.3)	23.4 (5.7)	24.1 (5.9)
Nulliparous (%)	42	23	41	54	62
College graduate (%)	64	71	68	65	59
Smoking habits (%)					
Never	69	74	70	66	65
Former	19	18	16	23	23
During pregnancy	11	8	14	11	12
Time of prenatal PFAS measurement (weeks gestation)^*c*^	9.6 (2.1)	9.9 (2.1)	9.7 (2.6)	9.4 (2.1)	9.4 (1.9)
Albumin (g/dL)	8.3 (2.3)	8.1 (2.2)	7.9 (2.2)	8.3 (2.0)	8.8 (2.4)
GFR (mL/min/1.73 m^2^)	101.9 (46.0)	102.1 (44.8)	107.9 (56.2)	97.3 (41.5)	99.9 (44.8)
Partner/household/neighborhood characteristics at enrollment
Individual-level household income > $70,000 (%)	61	54	64	66	66
Median household income in census tract ($)	51,798 (28,921)	51,816 (33,732)	51,772 (28,322)	55,625 (27,201)	51,681 (22,681)
Percent below poverty in census tract	7.4 (11.8)	9.0 (14.1)	7.1 (10.8)	6.6 (8.5)	6.6 (8.7)
Child characteristics
Female (%)	47	50	42	41	52
Race/ethnicity (%)^*d*^
White	59	55	63	67	64
Black	22	25	17	16	17
Hispanic	5	5	5	6	6
Asian	2	1	3	2	0
Other	11	13	12	9	12
Age at mid-childhood visit (years)	7.7 (1.0)	7.7 (1.1)	7.7 (1.1)	7.8 (0.9)	7.8 (1.0)
Mid-childhood cardiometabolic biomarkers
Leptin (ng/mL)	3.3 (3.9)	3.2 (3.4)	3.3 (4.8)	3.5 (3.0)	3.1 (5.0)
Adiponectin (μg/mL)	14.0 (9.7)	15.0 (11.0)	14.0 (9.5)	13.9 (9.5)	13.8 (7.9)
HOMA-IR	1.5 (1.3)	1.5 (1.1)	1.6 (1.7)	1.5 (1.3)	1.5 (1.5)
Abbreviations: BMI, body mass index; GFR, glomerular filtration rate; HOMA-IR, homeostatic model assessment of insulin resistance; IQR, interquartile range; PFAS, perfluoroalkyl substances; PFOA, perfluorooctanoate; Q, quartile. ^***a***^Missing data for participants overall (*n *= 665): 4 participants missing maternal prepregnancy BMI and education, 1 maternal smoking status, 160 albumin, 138 GFR, 67 individual-level household income, 8 census tract variables, 2 race/ethnicity, 6 age at mid-childhood visit, 52 leptin/adiponectin, 106 HOMA-IR. ^***b***^PFOA quartile maximum and minimum values: 0.9–4.1 ng/mL for Q1, 4.2–5.8 ng/mL for Q2, 5.9–7.9 ng/mL for Q3, and 8.0–22.4 ng/mL for Q4. ^***c***^Percent with prenatal PFAS measured in the second trimester: 11% for Q1, 13% for Q2, 9% for Q3, and 2% for Q4. ^***d***^Maternal race/ethnicity was substituted in 10% of children whose race/ethnicity was missing.					

Prenatal PFAS plasma concentrations in our cohort were typical for U.S. women during peak production, 1999–2000, and childhood PFAS plasma concentrations were similar to concentrations reported in U.S. children from 2007 through 2008 (CDC 2015) (Table 2). At both time points, highest concentrations were of PFOS although mid-childhood concentrations were substantially lower than prenatal concentrations [PFOS median (25th, 75th percentile) was 24.4 (17.9, 33.9) ng/mL in prenatal plasma and 6.2 (4.2, 9.7) ng/mL in childhood plasma]. Spearman correlations of PFAS concentrations in prenatal plasma were 0.24–0.72 and in mid-childhood plasma were 0.13–0.78. Correlations of the same PFASs measured in prenatal versus mid-childhood plasma were 0.08–0.40 (Table 2).

**Table 2:** PFAS plasma concentration distributions and Spearman correlation coefficients for PFAS with > 65% of detectable values.

Exposure	Prenatal (median, 9.6 weeks gestation)				Mid-childhood (median, 7.7 years)				
	PFOA	PFOS	PFNA	PFHxS	PFOA	PFOS	PFNA	PFHxS	PFDeA
PFAS plasma concentration (ng/mL)
Geometric mean (25th, 75th %ile)	5.3 (3.9, 7.6)	24.4 (17.9, 33.9)	0.6 (0.5, 0.9)	2.5 (1.6, 3.8)	4.2 (3.1, 6.0)	6.2 (4.2, 9.7)	1.7 (1.1, 2.3)	2.2 (1.2, 3.4)	0.3 (0.2, 0.5)
Minimum	0.9	4.6	< LOD (0.1)	< LOD (0.1)	< LOD (0.1)	< LOD (0.1)	< LOD (0.1)	< LOD (0.1)	< LOD (0.1)
Maximum	22.4	168.0	2.6	43.2	14.3	51.4	25.7	56.8	1.9
% below LOD	0	0	1.3	0.4	0.5	0.5	0.5	0.5	12
NHANES geometric mean	4.8^*a*^	28.0^*a*^	0.5^*a*^	1.8^*a*^	3.9^*b*^	11.3^*b*^	1.2^*b*^	2.4^*b*^	0.23^*b*^
Spearman correlation coefficients
Prenatal
PFOA	1.00
PFOS	0.72	1.00
PFNA	0.56	0.67	1.00
PFHxS	0.55	0.55	0.45	1.00
Mid-childhood
PFOA	0.15	0.10	0.08	0.18	1.00
PFOS	0.09	0.12	0.11	0.14	0.78	1.00
PFNA	0.11	0.10	0.08	0.07	0.43	0.34	1.00
PFHxS	0.12	0.12	0.07	0.40	0.59	0.66	0.13	1.00
PFDeA	0.10	0.13	0.11	0.08	0.69	0.59	0.55	0.34	1.00
Abbreviations: %tile, percentile; LOD: limit of detection; NHANES, U.S. National Health and Nutrition Examination Survey; PFAS, perfluoroalkyl substances; PFDeA, perfluorodecanoate; PFHxS, perfluorohexane sulfonate; PFNA, perfluorononanoate; PFOA, perfluorooctanoate; PFOS, perfluorooctane sulfonate. ^***a***^Women 1999–2000 (Calafat et al. 2007; CDC 2015). ^***b***^12- to 19-year-old children 2007–2008 (Calafat et al. 2007; CDC 2015).									

Mothers with higher PFOA concentrations during pregnancy were more likely to be younger, nulliparous, less educated, nonsmokers, to have had blood collection earlier in pregnancy, and to live in a census tract with lower percent below poverty, and their children were more likely to be white (Table 1). These associations were not consistent across all PFASs, with a markedly different pattern of associations for PFNA in our cohort, as described previously (Sagiv et al. 2015). Children with higher PFOA concentrations in mid-childhood were more likely to live in a census tract with higher median household income and lower percent below poverty, to be white and younger, and to have lower leptin and lower HOMA-IR (see Table S3). Mothers of children with higher PFOA concentrations were more likely to be older and college graduates.

### Prenatal PFAS Concentrations and Mid-Childhood Metabolic Profile

Prenatal PFAS plasma concentrations were not associated with leptin, adiponectin, or HOMA-IR in mid-childhood in unadjusted (data not shown) or covariate-adjusted (Table 3) analyses. For example, adjusted effect estimates were null for the associations of maternal PFOA concentrations with mid-childhood leptin (1.7% per IQR increment; 95% confidence interval (CI): –7.6, 12.0), adiponectin (1.2%; 95% CI: –7.2, 5.2), and HOMA-IR (–0.7%; 95% CI: –9.8, 9.4) (Table 3).

**Table 3:** Covariate-adjusted*^a^* associations of prenatal PFAS concentrations in maternal plasma in early pregnancy with cardiometabolic biomarkers in mid-childhood (median, 7.7 years of age).

PFAS/quartile	Leptin *n *= 484	Adiponectin *n *= 484	HOMA-IR *n *= 441
PFOA
IQR (3.8 ng/mL)	1.7 (–7.6, 12.0)	–1.2 (–7.2, 5.2)	–0.7 (–9.8, 9.4)
Q1 (0.9–4.1 ng/mL)	Reference	Reference	Reference
Q2 (4.2–5.8 ng/mL)	7.3 (–11.8, 30.6)	–13.3 (–23.6, –1.5)^*b*^	8.0 (–11.3, 31.3)
Q3 (5.9–7.9 ng/mL)	4.6 (–15.6, 29.7)	–2.1 (–14.8, 12.4)	4.4 (–15.3, 28.8)
Q4 (8.0–22.4 ng/mL)	3.0 (–17.4, 28.5)	–2.4 (–15.4, 12.6)	3.0 (–17.2, 28.1)
PFOS
IQR (16.0 ng/mL)	–0.3 (–7.7, 7.6)	1.1 (–3.8, 6.2)	–0.6 (–8.2, 7.6)
Q1 (4.6–18.8 ng/mL)	Reference	Reference	Reference
Q2 (18.9–25.5 ng/mL)	2.2 (–16.1, 24.5)	1.3 (–10.9, 15.2)	–12.2 (–27.7, 6.7)
Q3 (25.7–34.8 ng/mL)	5.1 (–15.0, 29.9)	0.8 (–12.2, 15.7)	–12.0 (–28.6, 8.5)
Q4 (34.9–168.0 ng/mL)	6.8 (–13.8, 32.3)	–2.2 (–14.9, 12.4)	1.6 (–17.9, 25.8)
PFNA
IQR (0.4 ng/mL)	0.5 (–8.8, 10.8)	–5.5 (–11.3, 0.6)	1.4 (–8, 11.7)
Q1 [< LOD (0.1)–0.40 ng/mL]	Reference	Reference	Reference
Q2 (0.50–0.60 ng/mL)	21.6 (–0.9, 49.2)	0.6 (–11.9, 14.9)	7.8 (–11.8, 31.9)
Q3 (0.70–0.90 ng/mL)	18.6 (–4.2, 46.9)	–8.2 (–20.1, 5.5)	13.1 (–8.6, 39.8)
Q4 (1.0–2.6 ng/mL)	5.1 (–17.6, 34.1)	–13.0 (–25.7, 1.9)	2.9 (–19.2, 31.1)
PFHxS
IQR (2.2 ng/mL)	–3.1 (–7.5, 1.5)	0.6 (–2.4, 3.6)	–2.0 (–5.9, 2.0)
Q1 [< LOD (0.1)–1.6 ng/mL]	Reference	Reference	Reference
Q2 (1.7–2.4 ng/mL)	13.5 (–7.3, 39)	–3.1 (–15.1, 10.6)	–6.7 (–23.7, 14.2)
Q3 (2.5–3.7 ng/mL)	13.8 (–7.6, 40.2)	–3.7 (–15.9, 10.3)	–13.5 (–29.6, 6.3)
Q4 (3.8–43.2 ng/mL)	2.8 (–16.2, 26)	–5.4 (–17.1, 8.1)	–17.1 (–32.3, 1.6)
Estimates are presented as percent change (95% confidence intervals) in outcome for *a*) concentration quartiles 2–4 versus quartile 1 and *b*) for each interquartile range increment in concentrations. Abbreviations: HOMA-IR, homeostatic model assessment of insulin resistance; IQR, interquartile range; LOD, limit of detection; PFAS, perfluoroalkyl substances; PFHxS, perfluorohexane sulfonate; PFNA, perfluorononanoate; PFOA, perfluorooctanoate; PFOS, perfluorooctane sulfonate; Q, quartile. ^***a***^Model adjusted for characteristics of child (age, sex, race/ethnicity), mother (age, education, parity, smoking during pregnancy), neighborhood census tract at enrollment (median household income, percent below poverty), and pregnancy hemodynamics (time of blood draw in weeks gestation). ^***b***^Estimate with 95% confidence intervals that do not cross the null.			

### Mid-Childhood PFAS Concentrations and Metabolic Profile

Mid-childhood PFAS concentrations were not associated with leptin or adiponectin measured at the same time in unadjusted (data not shown) or adjusted (Table 4) analyses, except for consistently lower leptin in children in higher quartiles (Q2–4) of PFOA plasma concentrations [versus the lowest quartile (Q1)].

**Table 4:** Covariate-adjusted*^a^* associations of mid-childhood PFAS plasma concentrations with cardiometabolic biomarkers at the same time (median, 7.7 years of age).

PFAS/quartile	Leptin *n *= 584	Adiponectin *n *= 584	HOMA–IR *n *= 541
PFOA
IQR (2.9 ng/mL)	–5.0 (–12.9, 3.6)	1.0 (–4.9, 7.4)	–10.1 (–17.3, –2.3)^*b*^
Q1 [< LOD (0.1)–3.0 ng/mL]	Reference	Reference	Reference
Q2 (3.1–4.3 ng/mL)	–17.2 (–31.6, 0.2)	16.3 (1.8, 32.9)^*b*^	–14.9 (–29.4, 2.6)
Q3 (4.4–6.0 ng/mL)	–23.3 (–37.0, –6.5)^*b*^	22.7 (6.9, 40.8)^*b*^	–27.3 (–40.0, –12.0)^*b*^
Q4 (6.1–14.3 ng/mL)	–20.1 (–35.1, –1.6)^*b*^	9.7 (–5.1, 26.8)	–25.3 (–38.7, –9.0)^*b*^
PFOS
IQR (5.5 ng/mL)	–5.2 (–11.4, 1.4)	–0.5 (–5.1, 4.3)	–10.1 (–16.4, –3.3)^*b*^
Q1 [< LOD (0.1)–4.2 ng/mL]	Reference	Reference	Reference
Q2 (4.2–6.2 ng/mL)	6.6 (–11.3, 28.1)	–1.3 (–13.3, 12.2)	3.4 (–13.6, 23.6)
Q3 (6.2–9.7 ng/mL)	–4.2 (–20.9, 16.1)	1.2 (–11.5, 15.8)	–12.9 (–27.6, 4.8)
Q4 (9.8–51.4 ng/mL)	–17.1 (–31.9, 0.8)	0.9 (–12, 15.8)	–24.7 (–37.8, –8.8)^*b*^
PFNA
IQR (1.2 ng/mL)	0.8 (–2.2, 4.0)	–2.1 (–4.2, 0.0)	–0.6 (–3.6, 2.6)
Q1 [< LOD (0.1)–1.0 ng/mL]	Reference	Reference	Reference
Q2 (1.1–1.5 ng/mL)	–13.9 (–28.1, 3.0)	–8.6 (–19.3, 3.5)	–25.0 (–37.0, –10.7)^*b*^
Q3 (1.6–2.3 ng/mL)	–6.6 (–23.2, 13.5)	7.5 (–6.1, 23.1)	–27.1 (–39.4, –12.1)^*b*^
Q4 (2.4–25.7 ng/mL)	–9.0 (–24.8, 10.1)	–9.1 (–20.4, 3.7)	–25.6 (–38.0, –10.7)^*b*^
PFHxS
IQR (2.2 ng/mL)	–0.3 (–2.6, 2.2)	0.3 (–1.4, 1.9)	–1.7 (–3.8, 0.5)
Q1 [< LOD (0.1)–1.1 ng/mL]	Reference	Reference	Reference
Q2 (1.2–1.9 ng/mL)	–4.3 (–20.3, 14.9)	–6.5 (–17.8, 6.2)	–5.1 (–20.9, 13.8)
Q3 (2.0–3.4 ng/mL)	–7.5 (–23.6, 11.9)	4.3 (–8.7, 19.1)	–6.7 (–22.7, 12.6)
Q4 (3.5–56.8 ng/mL)	–19.4 (–33.7, –2.1)^*b*^	3.4 (–9.8, 18.5)	–16.8 (–31.4, 0.8)
PFDeA
IQR (0.3 ng/mL)	–8.2 (–16.6, 0.9)	5.1 (–1.7, 12.3)	–14.7 (–22.1, –6.5)^*b*^
Q1 [< LOD (0.1)–0.2 ng/mL]	Reference	Reference	Reference
Q2 (≥ 0.3–< 0.4 ng/mL)	–9.3 (–24.4, 8.8)	6.1 (–6.5, 20.4)	–7.1 (–22.1, 10.6)
Q3 (≥ 0.4–< 0.5 ng/mL)	–8.2 (–24.2, 11.0)	18.0 (3.3, 34.7)^*b*^	–31.3 (–42.8, –17.5)^*b*^
Q4 (0.5–1.9 ng/mL)	–10.9 (–25.7, 6.9)	9.0 (–4.0, 23.7)	–21.5 (–34.0, –6.7)^*b*^
Estimates are presented as percent change (95% confidence intervals) in outcome for *a*) concentration quartiles 2–4 versus quartile 1 and *b*) for each interquartile range increment in concentration. Estimates with 95% confidence intervals that do not cross the null are bolded. Abbreviations: HOMA-IR, homeostatic model assessment of insulin resistance; IQR, interquartile range; LOD, limit of detection; PFAS, perfluoroalkyl substances; PFDeA, perfluorodecanoate; PFHxS, perfluorohexane sulfonate; PFNA, perfluorononanoate; PFOA, perfluorooctanoate; PFOS, perfluorooctane sulfonate; Q, quartile. ^***a***^Model adjusted for characteristics of child (age, sex, race/ethnicity), mother (age, education), and neighborhood census tract at mid-childhood (median household income, percent below poverty). ^***b***^Estimates with 95% confidence intervals that do not cross the null.			

Children with higher PFAS concentrations had lower HOMA-IR with weaker associations in covariate-adjusted [e.g., 10.1% lower HOMA-IR (95% CI: –17.3, –2.3) per IQR increment in PFOA; Table 4] versus unadjusted [e.g., 14.7% lower (95% CI –21.1, –7.8) per IQR increment in PFOA; data not shown] models. Strongest covariate-adjusted associations were with PFDeA [14.7% lower HOMA-IR (95% CI: –22.1, –6.5) per IQR increment] with weaker, imprecise effect estimates for PFHxS and PFNA (Table 4). Notably, HOMA-IR was monotonically lower across quartiles of PFOS and PFHxS concentrations, consistently lower in Q3 and Q4 versus Q1 of PFOA and PFDeA concentrations, and consistently lower in Q2–4 versus Q1 of PFNA concentration.

Covariate-adjusted penalized spline models of mid-childhood PFAS plasma concentrations and HOMA-IR were consistent with the quartile results. PFOS and PFHxS had an inverse dose–response association with HOMA-IR, and higher PFOA, PFNA, and PFDeA concentrations were also associated with lower HOMA-IR with possible thresholds of association. In all cases, effect estimates were imprecise in the higher PFAS concentration range where there were fewer values (see Figure S2).

### Effect Modification and Stratification by Sex

Child sex modified the associations between prenatal PFHxS concentration and HOMA-IR [*p* = 0.04; inverse association for boys and null for girls (data not shown)] and childhood PFOS concentration and HOMA-IR [*p* = 0.03; inverse association for girls and null for boys (see Table S4)]. Additionally, we observed sex-specific patterns in the associations between childhood PFAS concentrations and HOMA-IR in stratified analyses, even in cases where the interaction term was not significant. Specifically, childhood PFOA, PFOS, and PFDeA concentrations were inversely associated with HOMA-IR in girls with null associations in boys (see Table S4).

### Sensitivity Analyses

When we included all childhood PFAS plasma concentrations in the same covariate-adjusted model predicting HOMA-IR, PFDeA was the only PFAS significantly associated with HOMA-IR (data not shown). HOMA-IR was 14.5% (95% CI: –24.7, –2.9) lower per IQR increment in PFDeA.

When we examined the association between childhood PFAS plasma concentrations and HOMA-IR among only prepubertal participants [covariate-adjusted *n* = 386; median age (IQR), 7.6 (0.8) years], adjusted effect estimates were similar to those for the full cohort. For example, among prepubertal participants HOMA-IR was 9.3% (95% CI: –17.4, –0.5) lower per IQR increment in PFOA, 9.1% (95% CI: –16.0, –1.8) lower per IQR increment in PFOS, and 12.4% (95% CI: –20.8, –3.2) lower per IQR increment in PFDeA.

Linear and branched isomers of PFOA and PFOS measured in mid-childhood had similar patterns of association with mid-childhood metabolic profile as total concentrations of PFOA and PFOS [i.e., no association with leptin or adiponectin (data not shown) and inverse association with HOMA-IR]. HOMA-IR was 11.0% (95% CI: –18.3, –3.0) lower per IQR increment in n-PFOA, 9.5% (95% CI: –15.8, –2.7) lower per IQR increment in n-PFOS, and 12.3% (95% CI: –19.1, –5.0) lower per IQR increment in Sm-PFOS.

## Discussion

In a large, prospective Boston-area cohort, we found no evidence for an adverse association of prenatal or mid-childhood PFAS exposure with metabolic profile in children. Prenatal PFAS concentrations in early pregnancy were not associated with dysmetabolism in offspring in mid-childhood, and childhood PFAS concentrations were not contemporaneously associated with leptin or adiponectin. In fact, contrary to our *a priori* hypothesis, children with higher plasma concentrations of PFASs had lower HOMA-IR (i.e., less insulin resistance), with the strongest associations for PFDeA and in girls.

Our findings are biologically plausible given that PFASs may have a combination of detrimental and beneficial effects on metabolic status. For instance, PFASs are structurally similar to fatty acids, and *in vitro*, PFASs increase the expression of genes involved in fatty acid oxidation (Guruge et al. 2006; Hu et al. 2005). Up-regulation of fatty acid oxidation has been postulated to increase oxidative stress which, in turn, exacerbates insulin resistance (Karpe et al. 2011). On the other hand, but also based on their structural homology to fatty acids, PFASs activate nuclear PPAR-γ (Vanden Heuvel et al. 2006). Through PPAR-γ activation, PFAS exposure could improve insulin sensitivity by triggering the expression of genes that stimulate free fatty acid storage and thereby necessitate the use of glucose rather than fatty acids as a fuel substrate (Janani and Ranjitha Kumari 2015). Thiazolidinediones, PPAR-γ agonists used to treat type 2 diabetes, have been shown to lower serum insulin even in normal-weight individuals without diabetes (Yu et al. 2002). Also, recent cohorts have shown a protective association between PFAS exposure and neurocognitive/behavioral dysfunction (Power et al. 2013; Stein et al. 2013), hypothesized to result from PPAR-γ activation, although other studies (e.g., Hoffman et al. 2010) have shown null or direct associations.

As expected based on these contradictory mechanistic underpinnings, prior epidemiologic analyses have not shown a consistent association between PFAS exposure and insulin resistance in adult cohorts. In studies of the Canadian Health Measures Survey (Fisher et al. 2013) and U.S. National Health and Nutrition Examination Survey (Nelson et al. 2010), PFAS plasma concentrations were not associated with HOMA-IR. Similarly, in a cohort of ~ 55,000 U.S. adults with PFOA concentrations above background due to contaminated drinking water, there was no association between PFOA concentrations and diabetes, although when restricted to ~ 14,000 individuals with unchanged residential water district for > 20 years, higher PFOA was associated with decreased risk of diabetes (MacNeil et al. 2009). In two additional adolescent/adult cohorts (Lin et al. 2009; Lind et al. 2014), there were associations between some but not all PFASs and some but not all metabolic end points [e.g., association between PFNA, but not 6 other measured PFASs, and diabetes risk, but not HOMA-IR (Lind et al. 2014)].

Our study adds to the existing literature by examining PFAS exposure during fetal development and in childhood, life stages during which individuals are potentially more vulnerable to environmental insults (Symonds et al. 2009). Although our finding of no adverse effect of PFASs on metabolic status is in line with the cross-sectional adult studies, it is less consistent with the one prior prospective cohort study of prenatal PFAS exposure and offspring metabolic health in which higher maternal PFOA (but not PFOS, PFNA, or perfluorooctane sulfonamide) concentrations were associated with higher HOMA-IR, higher leptin, and lower adiponectin in female, but not male, offspring at 20 years of age (Halldorsson et al. 2012). In our cohort, we also found stronger associations in females, but associations were inverse and for childhood rather than prenatal PFAS concentrations. Individuals who are predisposed to develop insulin resistance due to male sex (Friend et al. 2013) may do so regardless of any potential effect of PFASs, although these sex-stratified findings require replication.

Our findings are also partly inconsistent with a cross-sectional study of 8- to 10-year-old Danish children in which PFOA and PFOS plasma concentrations were associated with higher HOMA-IR, but not leptin or adiponectin, in overweight, but not normal-weight children (Timmermann et al. 2014). In the present study, we opted not to assess for effect modification by child weight because of the possibility that weight is on the causal pathway and potential for collider bias (Cole et al. 2010).

We recognize that our finding of an inverse association between PFAS concentrations and insulin resistance in childhood could be attributable to chance or residual confounding. We performed a large number of analyses, and multiple testing could have led to statistically significant associations by chance, although consistent patterns in our results suggests against this possibility. Additionally, residual negative confounding could have occurred by socioeconomic status (SES) which is directly associated with PFAS concentrations (see Table S3) and inversely associated with insulin resistance in Project Viva (data not shown). However, we accounted for several individual (race/ethnicity, education) and census-tract (median household income, percent below poverty) markers of SES in covariate-adjusted analyses presented here, and we saw no attenuation of results when we additionally controlled for individual-level household income or SES-related behavioral determinants of metabolic health (fast food intake, soda intake, physical activity, and screen time). We also considered the possibility that because insulin resistance increases during puberty (Vryonidou et al. 2015), an association between PFAS concentrations and delayed puberty (Lopez-Espinosa et al. 2011) could have driven the inverse association between childhood PFAS concentrations and HOMA-IR. However, when we restricted analyses to prepubertal children, effect estimates were similar to those obtained from the full cohort, suggesting against this possibility. Additional, well-controlled studies of early-life PFAS exposure in postpubertal children and adults will help to confirm the magnitude and directionality of the association with metabolic status.

In our cohort, PFDeA had a stronger inverse association with HOMA-IR than concentrations of the other PFASs, in both individual- and multi-pollutant models. However, our study was limited based on our inability to conduct analyses of prenatal PFDeA due to the large number of plasma samples with concentrations below the LOD. In addition, childhood PFDeA plasma concentration had relatively low variability. Few human health studies have evaluated PFDeA, and additional research would increase our understanding of the potential role of PFDeA on health outcomes. Also, in our study, plasma concentrations of several PFASs were nonmonotonically associated with lower HOMA-IR. Attention to the pattern of PFAS associations with health outcomes in future studies would help to elucidate whether a threshold effect exists.

Generalizability is a limitation of Project Viva because our cohort consists of primarily white children of moderately high SES. Strengths of the study include use of a large, prospective cohort with multiple potential confounding variables, measurement of plasma concentrations of several PFASs at different time points, and biochemical measures of metabolic function in childhood.

In summary, we found no evidence for an adverse effect of early-life PFAS exposure on metabolic function in mid-childhood. In our cohort, children with higher PFAS plasma concentrations had lower insulin resistance. Although this finding is biologically plausible, it is in contrast to the existing limited literature on early-life PFAS exposure and would benefit from replication.

## Supplemental Material

(505 KB) PDF
