# Supplementary material for: Early-Life Exposure to Perfluoroalkyl Substances and Childhood Metabolic Function
Source: Environ Health Perspect. 2016 Sep 2;125(3):481–7. doi: 10.1289/EHP303 (PMC5332186; doi:10.1289/EHP303)
Supplement: (505 KB) PDF [file EHP303.s001.acco.pdf]

Environ Health Perspect
DOI: 10.1289/EHP303

Note to readers with disabilities: *EHP* strives to ensure that all journal content is accessible to all readers. However, some figures and Supplemental Material published in *EHP* articles may not conform to 508 standards due to the complexity of the information being presented. If you need assistance accessing journal content, please contact ehp508@niehs.nih.gov. Our staff will work with you to assess and meet your accessibility needs within 3 working days.

Supplemental Material

Early Life Exposure to Perfluoroalkyl Substances and Childhood Metabolic Function

Abby F. Fleisch, Sheryl L. Rifas-Shiman, Ana M. Mora, Antonia M. Calafat, Xiaoyun Ye, Heike Luttmann-Gibson, Matthew W. Gillman, Emily Oken, and Sharon K. Sagiv

Table of Contents

Table S1: Characteristics of Project Viva participants in the full cohort and in the subset not included versus included in analyses

Table S2: Distributions of child plasma concentrations of linear and branched isomers of PFOA and PFOS with greater than 65% of detectable values

Table S3: Characteristics of Project Viva participants by PFOA plasma concentration in mid-childhood (n=643 in analytic dataset with measurement of PFOA in mid-childhood)

Table S4: Covariate-adjusted associations of mid-childhood PFAS plasma concentration (median: 7.7 years of age) with ln-transformed HOMA-IR measured at the same time in female versus male children. Estimates are presented as percent change (95% confidence intervals) in outcome for (1) concentration quartiles 2-4 versus quartile 1 and (2) for each interquartile range increment in concentration. Estimates with 95% confidence intervals that do not cross the null are bolded.

Figure S1: Project Viva cohort sample size and participation. 665 participants had data available for at least one exposure and one outcome studied.

Figure S2: Covariate-adjusted associations of mid-childhood PFAS plasma concentrations and HOMA-IR measured at the same time (median: 7.7 years of age) modeled using penalized splines. Maximum and minimum values for each PFAS quartile are overlaid on the spline graphs.

Table S1. Characteristics of Project Viva participants in the full cohort and in the subset not included versus included in analyses.

	Full cohort (n=2,128)		Subset not included in analyses (n=1,463)		Subset included in analyses (n=665)	
	**N**	**Median (IQR) or %**	**N**	**Median (IQR) or %**	**N**	**Median (IQR) or %**
**Maternal characteristics**						
Age at enrollment (years)	2,128	32.2 (6.4)	1,463	32.1 (6)	665	32.5 (7.1)
Prepregnancy BMI (kg/m^2)	2,112	23.5 (6.4)	1,451	23.3 (6.4)	661	23.7 (5.9)
Nulliparous (%)	2,128	48	1,463	50	665	42
College graduate (%)	2,104	65	1,443	65	661	64
Smoking habits (%)	2,107		1,443		664	
Never		68		68		69
Former		19		19		19
During pregnancy		13		13		11
Maternal PFAS plasma concentration (ng/mL)	1,645		1,109		536	
PFOA		5.8 (3.8)		6 (3.7)		5.4 (3.8)
PFOS		25.7 (16.0)		26.3 (16.1)		24.4 (16.1)
PFNA		0.7 (0.4)		0.7 (0.5)		0.6 (0.4)
PFHxS		2.4 (2.2)		2.5 (2.1)		2.4 (2.2)
Time of maternal PFAS measurement (weeks gestation)	1,168	9.7 (2.3)	1,122	9.7 (2.3)	546	9.6 (2.1)
**Partner/ Household/Neighborhood characteristics at enrollment**						
Median household income in census tract ($)	2,108	53,934 (26,821)	1,451	54,688 (26,483)	657	51,798 (28,921)
Percent below poverty in census tract (%)	2,108	7.1 (10.6)	1,451	6.8 (9.9)	657	7.4 (11.8)
**Child characteristics**						
Female (%)	2,128	48	1,463	49	665	47
Race/ethnicity (%)[c]	2,109		1,446		663	

White		64		66		59
Black		17		15		22
Hispanic		6		6		5
Asian		5		5		2
Other		9		8		11
Age at mid-childhood visit (years)	1,116	7.7 (1.1)	457	7.7 (1.1)	665	7.7 (1.0)

Abbreviations: BMI: body mass index; interquartile range; PFAS: perfluoroalkyl substances; PFOA: perfluorooctanoate; PFOS: perfluorooctane sulfonate; PFNA: perfluorononanoate; PFHxS: perfluorohexane sulfonate; PFDeA: perfluorodecanoate

Table S2. Distributions of child plasma concentrations of linear and branched isomers of PFOA and PFOS with greater than 65% of detectable values

**Exposure**	n-PFOA	n-PFOS	Sm-PFOS
**N**	643	643	643
**Geometric mean (25th, 75th %ile)**	4.0 (3.0, 5.7)	4.5 (3.0, 7.1)	1.7 (1.1, 2.8)
**Minimum**	0.1	0.1	0.1
**Maximum**	13.8	34.2	16.8
**% below LOD**	0.5	0.5	0.5

Abbreviations: PFOA: perfluorooctanoate; PFOS: perfluorooctane sulfonate; n-PFOA: linear perfluorooctanoate; n-PFOS: linear perfluorooctane sulfonate; Sm-PFOS: sum of perfluoromethylheptane sulfonates; LOD: limit of detection

Table S3. Characteristics of Project Viva participants by PFOA plasma concentration in mid-childhood (n=643 in analytic dataset with measurement of PFOA in mid-childhood)[a]

	Quartiles[b] of child PFOA			
	Q1 (lowest) n=160	Q2 n=160	Q3 n=164	Q4 (highest) n=159
	Median (IQR) or %			
**Maternal characteristics**				
Age at enrollment (years)	30.8 (10.6)	32.9 (7.2)	32.7 (5.9)	33.1 (6.1)
College graduate (%)[c]	42	65	71	79
**Household/Neighborhood characteristics**				
Median household income in census tract ($)[d]	50,305 (26,716)	60,414 (29,418)	61,231 (23,855)	68,533 (29,799)
Percent below poverty in census tract (%)[d]	7.4 (13.0)	4.7 (7.4)	4.2 (3.9)	3.7 (3.6)
**Child characteristics**				
Female (%)	47	49	44	48
Race/ethnicity (%)[e]				
White	28	60	66	82
Black	45	20	15	7
Hispanic	9	7	5	1
Asian	4	2	2	3
Other	14	11	12	8
Age at mid-childhood visit (years)	8.2 (1.7)	7.7 (1.1)	7.7 (0.7)	7.6 (0.6)
Leptin (ng/mL)	4.3 (6.7)	3.3 (3.9)	2.6 (3.6)	3.1 (3.0)
Adiponectin (µg/mL)	12.1 (11.1)	14.6 (8.6)	15.0 (8.7)	14.3 (8.5)
HOMA-IR	1.8 (1.9)	1.6 (1.1)	1.3 (1.4)	1.3 (1.2)

Abbreviations: HOMA-IR: homeostatic model assessment of insulin resistance; IQR: interquartile range; PFOA: perfluorooctanoate; Q1: quartile 1; Q2: quartile 2; Q3: quartile 3; Q4: quartile 4

[a]Missing data as follows: 4 participants missing maternal education, 6 census tract variables, 2 race/ethnicity, 50 leptin, 50 adiponectin, 92 HOMA-IR

[b]PFOA maximum and minimum values: < LOD (0.1)-3.0 ng/mL for Q1, 3.1-4.3 ng/mL for Q2, 4.4-6.0 ng/mL for Q3, and 6.1-14.3 ng/mL for Q4

[c]At time of enrollment (median 9.6 weeks gestation)

[d]At time of mid-childhood follow-up visit (median 7.7 years)

[e]Maternal race/ethnicity was substituted in 10% of children whose race/ethnicity was missing.

Table S4. Covariate-adjusted[a] associations of mid-childhood PFAS plasma concentration (median: 7.7 years of age) with ln-transformed HOMA-IR measured at the same time in female versus male children.

	**Females n=257**	**Males n=284**
	Estimate (95% CI) for percent change in HOMA-IR	
**PFOA**		
IQR (2.9 ng/mL)	**-15.6 (-25.4, -4.6)**	-6.1 (-16.2, 5.2)
Q1 [<LOD (0.1)-3.0 ng/mL]	Reference	Reference
Q2 (3.1-4.3 ng/mL)	**-28.6 (-45.6, -6.2)**	-5.0 (-26.8, 23.2)
Q3 (4.4-6.0 ng/mL)	**-32.8 (-49.7, -10.3)**	**-25.3 (-42.4, -3.0)**
Q4 (6.1-14.3 ng/mL)	**-39.5 (-54.7, -19.3)**	-13.1 (-34.1, 14.6)
**PFOS**		
IQR (5.5 ng/mL)	**-16.7 (-25.7, -6.7)**	-6.2 (-14.7, 3.1)
Q1 [<LOD (0.1)-4.2 ng/mL]	Reference	Reference
Q2 (4.2-6.2 ng/mL)	5.9 (-18.2, 37.0)	0.4 (-21.7, 28.8)
Q3 (6.2-9.7 ng/mL)	-11.4 (-32.5, 16.3)	-17.4 (-36.1, 6.7)
Q4 (9.8-51.4 ng/mL)	**-30.7 (-47.5, -8.4)**	-21.9 (-40.1, 1.8)
**PFNA**		
IQR (1.2 ng/mL)	-2.7 (-7.3, 2.1)	1.0 (-3.0, 5.2)
Q1 [<LOD (0.1)-1.0 ng/mL]	Reference	Reference
Q2 (1.1-1.5 ng/mL)	**-34.0 (-49.0, -14.6)**	-18.3 (-35.6, 3.7)
Q3 (1.6-2.3 ng/mL)	**-25.5 (-43.9, -1.0)**	**-30.4 (-45.8, -10.6)**
Q4 (2.4-25.7 ng/mL)	**-32.8 (-48.6, -12.1)**	-15.8 (-34.4, 8.2)
**PFHxS**		
IQR (2.2 ng/mL)	-1.8 (-5.1, 1.6)	-1.9 (-4.6, 0.9)
Q1 [<LOD (0.1)-1.1 ng/mL]	Reference	Reference
Q2 (1.2-1.9 ng/mL)	-11.7 (-32, 14.6)	-3.1 (-25, 25.2)
Q3 (2.0-3.4 ng/mL)	-22.2 (-40.6, 1.9)	7.6 (-17.7, 40.5)
Q4 (3.5-56.8 ng/mL)	-24.6 (-43.6, 0.8)	-13.5 (-33.3, 12.1)
**PFDeA**		
IQR (0.3 ng/mL)	**-22.2 (-32.0, -11.1)**	-8.3 (-19.1, 4.0)
Q1 [<LOD (0.1)-0.2 ng/mL]	Reference	Reference
Q2 (≥0.3-<0.4 ng/mL)	-10.6 (-31.4, 16.6)	-10.0 (-29.0, 14.1)
Q3 (≥0.4-<0.5 ng/mL)	**-32.5 (-48.2, -11.9)**	**-32.6 (-47.9, -12.7)**
Q4 (0.5-1.9 ng/mL)	**-36.0 (-50.4, -17.4)**	-7.9 (-27.5, 17.0)

Estimates are presented as percent change (95% confidence intervals) in outcome for (1) concentration quartiles 2-4 versus quartile 1 and (2) for each interquartile range increment in concentrations. Estimates with 95% confidence intervals that do not cross the null are bolded. Abbreviations: HOMA-IR: homeostatic model assessment of insulin resistance; IQR: interquartile range; LOD: limit of detection; PFAS: perfluoroalkyl substances; PFOA: perfluorooctanoate; PFOS: perfluorooctane sulfonate; PFNA: perfluorononanoate; PFHxS: perfluorohexane sulfonate; PFDeA: perfluorodecanoate; Q1: quartile 1; Q2: quartile 2; Q3: quartile 3; Q4: quartile 4

[a]Model adjusted for characteristics of child (age, race/ethnicity), mother (age, education), and neighborhood census tract at mid-childhood (median household income, percent below poverty)

Figure S1. Project Viva cohort sample size and participation. 665 participants had data available for at least one exposure and one outcome studied.

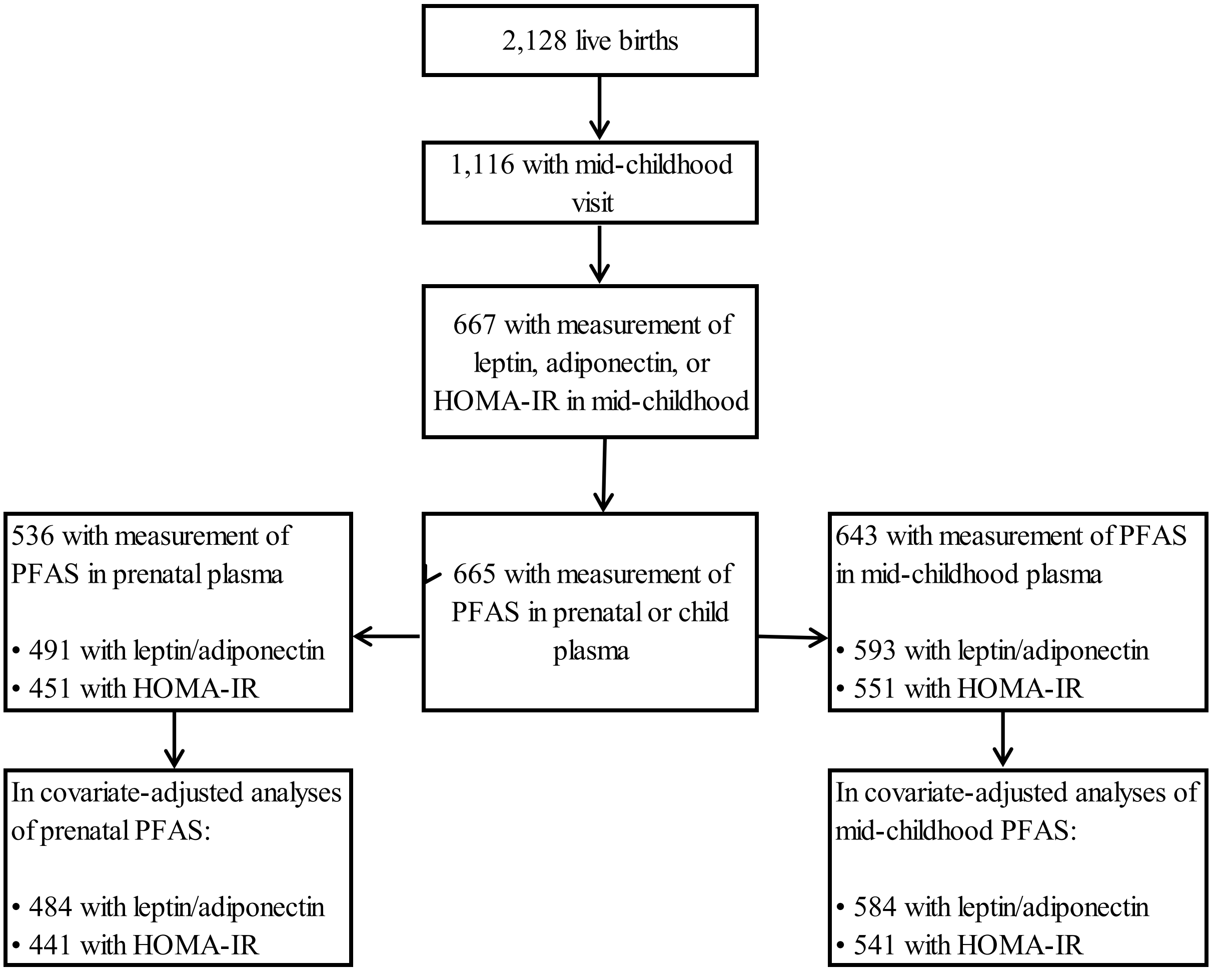

Figure S2. Covariate-adjusted[a] associations of mid-childhood PFAS plasma concentrations and HOMA-IR measured at the same time (median: 7.7 years of age) modeled using penalized splines. Maximum and minimum values for each PFAS quartile are overlaid on the spline graphs.

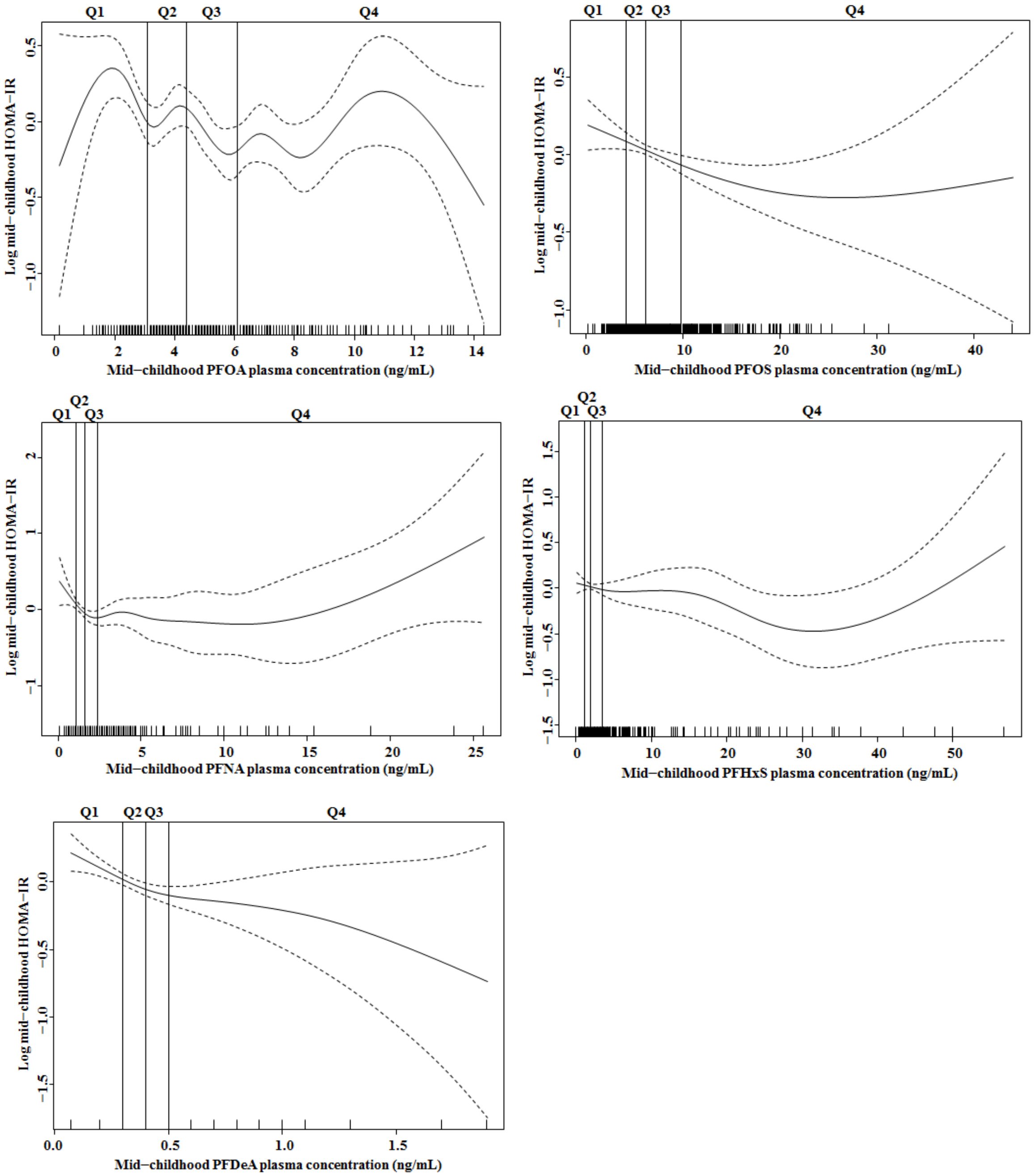

Abbreviations: HOMA-IR: homeostatic model assessment of insulin resistance; PFAS: perfluoroalkyl substances; PFOA: perfluorooctanoate; PFOS: perfluorooctane sulfonate; PFNA: perfluorononanoate; PFHxS: perfluorohexane sulfonate; PFDeA: perfluorodecanoate; Q1: quartile 1; Q2: quartile 2; Q3: quartile 3; Q4: quartile 4

[a]Model adjusted for characteristics of child (age, sex, race/ethnicity), mother (age, education), and neighborhood census tract at mid-childhood (median household income, percent below poverty)
